# Pretreatment of *Gymnema sylvestre* revealed the protection against acetic acid-induced ulcerative colitis in rats

**DOI:** 10.1186/1472-6882-14-49

**Published:** 2014-02-10

**Authors:** Abdulaziz M Aleisa, Salim S Al-Rejaie, Hatem M Abuohashish, Mohammed S Ola, Mihir Y Parmar, Mohammed M Ahmed

**Affiliations:** 1 Department of Pharmacology and Toxicology, College of Pharmacy, King Saud University, P.O. Box 2457, Riyadh 11451, Saudi Arabia; 2 Department of Biomedical Dental Sciences, College of Dentistry, Dammam University, Dammam 31441, Saudi Arabia; 3 Department of Biochemistry, College of Science, King Saud University, Riyadh 11415, Saudi Arabia

**Keywords:** *Gymnema sylvestre*, Inflammatory bowel diseases, Oxidative stress, Ulcerative colitis

## Abstract

**Background:**

Overproduction of free radicals and decreased antioxidant capacity are well-known risk factors for inflammatory bowel diseases. *Gymnema sylvestre* (*GS*) leaves extract is distinguished for its anti-diabetic, antioxidant and anti-inflammatory properties. Present study is designed to evaluate the preventative activities of *GS* against acetic acid (AA)-induced ulcerative colitis in Wistar rats.

**Methods:**

Experimentally ulcerative colitis (UC) was induced by AA in animals pretreated with three different doses of *GS* leaves extract (50, 100, 200 mg/kg/day) and a single dose of mesalazine (MES, 300 mg/kg/day) for seven days. Twenty four hours later, animals were sacrificed and the colonic tissues were collected. Colonic mucus content was determined using Alcian blue dye binding technique. Levels of thiobarbituric acid reactive substances (TBARS), total glutathione sulfhydryl group (T-GSH) and non-protein sulfhydryl group (NPSH) as well as the activity of the antioxidant enzymes superoxide dismutase (SOD) and catalase (CAT) were estimated in colon tissues. Colonic nucleic acids (DNA and RNA) and total protein (TP) concentrations were also determined. Levels of pro-inflammatory cytokines including interleukin-1 beta (IL-1β), tumor necrosis factor-alpha (TNF-α) and interleukin-6 (IL-6) as well as prostaglandin E_2_ (PGE_2_) and nitric oxide (NO) were estimated in colonic tissues. The histopathological changes of the colonic tissues were also observed.

**Results:**

In AA administered group TBARS levels were increased, while colonic mucus content, T-GSH and NP-SH, SOD and CAT were reduced in colon. Pretreatment with *GS* inhibited TBARS elevation as well as mucus content, T-GSH and NP-SH reduction. Enzymatic activities of SOD and CAT were brought back to their normal levels in *GS* pretreated group. A significant reduction in DNA, RNA and TP levels was seen following AA administration and this inhibition was significantly eliminated by *GS* treatment. *GS* pretreatment also inhibited AA-induced elevation of pro-inflammatory cytokines, PGE_2_ and NO levels in colon. The apparent UC protection was further confirmed by the histopathological screening.

**Conclusion:**

The *GS* leaves extract showed significant amelioration of experimentally induced colitis, which may be attributed to its anti-inflammatory and antioxidant property.

## Background

Ulcerative colitis and Crohn’s disease (CD) are mutually known as inflammatory bowel disease (IBD). Epidemiologically, IBD are heterogenic distributed disorder around the world [1]. In the United States and Western communities the incidence of UC is 7%, with a peak incidence between ages 20 and 25 years. Several etiological factors were suggested to be implicated in the pathogenesis of UC including genetic, immunological, and environmental factors. However, the exact pathophysiology of the disease is still unclear [2].

Inflammatory changes associated with UC are limited to the mucosa and typically affects the rectum but often extends to involve the whole colon [3,4]. Both excessive inflammation and oxidative stress play a pivotal role in the pathogenesis of UC [5,6]. The pathophysiology of UC as an inflammatory disease mainly characterized by migration of neutrophils, basophils and other leukocytes to the mucosa membranes and the superficial ulcers [7]. This process leads to release of inflammatory mediators such as cytokines and arachidonic acid metabolites, as well as free radicals resulting in oxidative damage to the colonic tissue [8,9]. Studies demonstrated that oxidative injury resulting from free radicals overproduction such as reactive oxygen species (ROS) and reactive nitrogen species (RNS) in patients with colitis can lead to adverse effects such as lipid peroxidation (LPO) of the cellular membrane and attack on tissue proteins and nucleic acids [10]. Generation of ROS and the subsequent LPO reduces cellular antioxidant capacity, which leads to prominent colonic inflammation. Management of UC using therapies with limited toxicity is a severe challenge.

The commonly used medical treatments for UC include 5-aminosalicylic acid, corticosteroids, azathioprine, 6-mercaptopurine, methotrexate, cyclosporine, antibiotics (e.g., metronidazole, ciprofloxacin, and vancomycin), and the TNF antagonist, infliximab [11]. These medications lack specificity and are linked with numerous side effects. Therefore, there is an urgent need to develop an effective and safe treatments and therapeutic approaches to treat such a disease in order to improve quality of life and psychosocial functioning of patients.

Several medicinal plants are characterized by their antioxidant and anti-inflammatory properties. In the study we focused on utilizing *GS* R. Br. a well-known medicinal plant from *Asclepiadaceae* family which is widely distributed in Southern India, tropical Africa and Australia, where it has been used traditionally as a folklore medicine [12]. Previous studies showed medical benefits of *GS* in improving urination, stomach stimulation, and diabetes [13-15]. *GS* leaves contains a group of triterpenoids and saponins known as gymnemic acids [12,16], alkaloids, acidic glycosides and anthroquinones and their derivatives [17]. These active constituents were found to promote ulcer healing by forming protective mucus barrier [18]. As shown in earlier studies, overproduction of ROS and inflammation plays an important role in the pathogenesis of UC, leading to oxidative damage in colonic tissues [5,19,20]. With respect to the high antioxidant capacity and anti-inflammatory activity, *GS* would be expected to reduce injury and/or improve tissue healing following injury from ulcerative colitis. In the present study, the preventative properties of *GS* leaves extract was evaluated by measuring potential pro oxidative and inflammatory markers known to damage the tissue in experimental model of UC by AA in Wistar rats.

## Methods

### Animals

The present study was conducted using 12 weeks old male Wister albino rats weighting 250–280 g. Animals were supplied by the Experimental Animal Care Center, College of Pharmacy, King Saud University, Riyadh, Saudi Arabia. Controlled environmental conditions (25°C and a 12 h light/dark cycle) were provided to the animals, which had a free access to Purina rat chow (Manufactured by Grain Silos and Flour Mills Organization, Riyadh, Saudi Arabia) and tap water. Animal experiments were conducted after official approval by following the guidelines of the Ethics Committee of the Experimental Animal Care Center, College of Pharmacy, King Saud University, Riyadh, Saudi Arabia.

### Plant extract

*GS* leaves dried ethanolic extract filled in capsules (200 mg in each) with the brand name "Diaglu" manufactured by MEPACO, Egypt and the recommended therapeutic dose was one capsule twice a day as dietary supplement. The extract used in present study was standardized as 25% gymneric acids as major constituents besides there are anthroquinones and their derivatives present in the extract. The dried powder was suspended in 0.25% carboxymethyl cellulose (CMC) solution and administered orally (gavage) in the doses of 50, 100 and 200 mg/kg body weight to fasted Wistar rats. The three doses of the extract have taken to find the dose dependent effect.

### Phytochemical analysis

*GS* dried ethanol leaves extract was screened for its phytochemical constituents using Agilent 6410 Triple Quadrupole Mass Spectrometer (Agilent Technologies, Santa Clora, CA, USA), which was equipped with an electrospray ionization interface coupled to an Agilent 1200 HPLC (Agilent Technologies, Santa Clora, CA, USA). Direct injection of the samples was allowed by a connector instead of the column. Two solvents were in the mobile phase: (A) HPLC grade water and (B) acetonitrile (ACN), which were mixed in 1:1 ratio. For mass spectrometry (MS), test solutions were prepared by diluting the stock solutions with ACN/H_2_O mixture. Using a flow rate of 0.4 mL/min and a run time of 3 min, 10 μL from each sample was injected into the LC-MS/MS. MS parameters were optimized for scan mode. The mass range of m/z 650–850 was used to perform MS2 scans for mass signals screening of the different compounds.

### Chemicals

Mesalazine was purchased from Shire pharmaceuticals Inc. USA. Thiobarbituric acid reactive substances (TBARS) assay kit was purchased from ZeptoMetrix Inc, USA. Tumor necrosis factor-α (TNF-α), interleukin-1β (IL-1β), interleukin-6 (IL-6), prostaglandin E_2_ (PGE_2_) and nitric oxide (NO) kits were purchased from R&D systems Inc, USA. All other chemicals used were of analytical reagent grade.

### Experimental design

Animals were randomly allocated into seven groups (six animals in each) as follows: Control (Cont), AA treated rats, *GS* 200 mg/kg/day, *GS* 50 mg/kg/day + AA, *GS* 100 mg/kg/day + AA, *GS* 200 mg/kg/day + AA and MES 300 mg/kg/day + AA. *GS* dried ethanol leaves extract and MES were treated to the rats for 7 consecutive days by gavage [21]. At the 7th day of treatment, UC was induced in all AA groups. 24 hr later, animals were sacrificed under deep anesthesia [22] and 5–6 cm of the colon specimens were dissected, washed with saline solution and weighted. A small cross section of colon tissue from each group was fixed in 10% formaldehyde solution for histopathological screening. The remaining colonic tissues were kept at -75°C (Ultra-low freezer, Environmental Equipment, Cincinnati, Ohio, USA) for biochemical analysis.

### Induction of UC in rats

The experimental induction of UC was performed in accordance with Mousavizadeh et al. method [23]. Using a 2.7 mm soft pediatric catheter, animals were trans-rectally administered 2 mL of 4% AA solution (v/v; Merck, Darmstadt, Germany) under light ether anesthesia. To avoid AA leakage, rats were then holed horizontally for 2 minutes. Exactly similar procedure was performed to control animals using equal volume of normal saline instead of AA solution.

### Estimation of the adherent colonic mucus

The method described by Popov et al. [24] was used to estimate the colon adherent mucus concentration. A small sections from animals colonic tissues were transferred immediately after weighing to a solution of 1% Alcian blue and 0.16 mol/L sucrose solution (pH 5) for 24 hr. The sucrose solution was used to rinse the excess dye. The complexed dye with the colonic wall mucus was extracted using 0.5 mol/L MgCl2 solution. Then, the blue extract was mixed with diethyl ether in a ratio of 1:1. After centrifugation at 4000 RPM, the absorbance of the aqueous layer was measured at 580 nm. The quantity of Alcian blue extracted in μg/grams of the wet colon was then calculated.

### Estimation of TBARS levels in colon

The LPO product malondialdehyde (MDA) was estimated by using an assay kit of TBARS in colon tissue (ZeptoMetrix Inc, USA). In brief, 100 μL of the colon tissue homogenate was mixed with 2.5 mL the kit reaction buffer then heated for 1 hr at 95°C. After cooling, the absorbance of the supernatant was measured at 532 nm. The LPO products are expressed in terms of nmoles MDA/mg protein.

### Estimations of T-GSH and NPSH concentrations in colon

The procedure described by Sedlak et al. [25] was used to determine colonic concentration of T-GSH and NP-SH. For the T-GSH estimation, 0.5 mL of the cold 0.02 mol/L EDTA colon tissues homogenate was added to 0.2 mol/L Tris buffer (pH 8.2) and 0.1 mL of 0.01 mol/L Ellman’s reagent, [5,5’-dithiobis-(2-nitr-benzoic acid)] (DTNB). Then samples were centrifuged at 3000 rpm for 15 min. The absorbance of the clear supernatant was measured at 412 nm by using spectrophotometer (LKB-Pharmacia, Mark II, Ireland). For NP-SH estimation, the homogenate was mixed with 1 mL of 50% trichloroacetic acid (TCA). Samples were then shaken intermittently for l0-15 min and centrifuged for 15 min at 3000 rpm. In a ratio of 1:2 the supernatant was mixed with 0.4 mol/L Tris buffer (pH 8.9) then 0.1 mL DTNB was added. The absorbance of the mixture was read within 5 min at 412 nm.

### Estimation of SOD and CAT activities in colon

The enzymatic activity of the antioxidant enzyme SOD was measured in post-mitochondrial supernatant of the colon homogenate by Kono [26] method. Superoxide anions generated hydroxylamine hydrochloride oxidation mediate nitro-bluetetrazolium reduction to a blue formazon, which was then measured at 560 nm under aerobic conditions. Superoxide dismutase inhibits nitrobluetetrazolium reduction. The extent of the inhibition was taken as a measure of SOD activity and expressed as units/mg protein. The CAT enzymatic activity was measured by using Aebi, [27] method. The post-mitochondrial supernatant of the colon homogenate was mixed with 50 mmol/L phosphate buffer (pH 7.0) and 20 mmol/L H_2_O_2_. The enzymatic activity CAT was determined following the decrease in absorbance at 240 nm and expressed in terms of units/mg protein.

### Determination of nucleic acids and total protein (TP) levels in colon

The concentrations of nucleic acids (DNA and RNA) in colon tissues were measured in accordance with the method described by Bregman [28]. The homogenized colon tissues were suspended in 5 mL of 10% ice-cold trichloroacetic acid (TCA). Samples were centrifuged and the resulted pellets were extracted with 95% ethanol twice. The nucleic acids content was extracted in 5% TCA. For DNA determination, extracts were treated with diphenylamine reagent and the resulted blue color was measured at 600 nm. For RNA quantification, the extracts were treated with orcinol reagent and the intensity of the green color was measured at 660 nm. Total protein in colon tissues were estimated by using Lowry [29] method following Bovine plasma albumin as a standard.

### Determination of inflammatory cytokines, PGE_2_ and NO levels in colon tissues

Animals colon levels of pro-inflammatory cytokines including IL-1β, IL-6 and TNF-α, and PGE_2_ were determined by using enzyme-linked immunoabsorbent assay ELISA kits (R&D systems Inc, USA) in accordance with Mousavizadeh et al. [23] method. The results were expressed as pg/mg tissue. The NO concentrations in colonic tissues were estimated by Griess reaction method using commercial kit (R&D systems Inc, USA).

### Histopathological assessment of colitis

Cross section of colon tissues were fixed in 10% formaldehyde solution then embedded into paraffin wax blocks and cut using a microtome. Samples were stained with haematoxylin and eosin stain (H&E), mounted and observed microscopically by a histopathologist in blinded fashion. Histopathological slides were screened for mucosal ulceration, hyperemia, necrosis, edema, cellular infiltrate and goblet cell hyperplasia.

### Statistical analysis

Experimental data were expressed as means ± SEM Statistical analysis was carried out using one-way ANOVA followed by Newman-Keuls as post hoc test. P values of ≤ 0.05 were considered statistically significant. All statistical analysis was conducted by using Graph Pad Prism (version 5) software.

## Results

### Phytochemical constituents of *GS*

Phytochemical screening of the dried ethanol *GS* leaves extract revealed the presence of several gymnemic acids with different molecular weights, which are summarized in Table 1.

**Table 1:** **List of compounds found in the negative MS scan spectra of the dried ethanol *GS *** **leaves extract**

**No.**	**Name of compound**	**Molecular weight**
**A**	Gymnemic acid I	807
**B**	Gymnemic acid II	809
**C**	Gymnemic acid III	809
**D**	Gymnemic acid IV	807
**E**	Gymnemic acid V	807
**F**	Gymnemic acid VII	666
**G**	Gymnemic acid XI	807
**H**	Gymnemic acid XIII	766
**I**	Gymnemic acid XVII	786
**J**	Gymnemic acid XVIII	786
**K**	Deacylgymnemic acid	682
**L**	Gymnemasin B	828

### Effect of *GS* on colonic weights and mucus content

**Figure 1:**
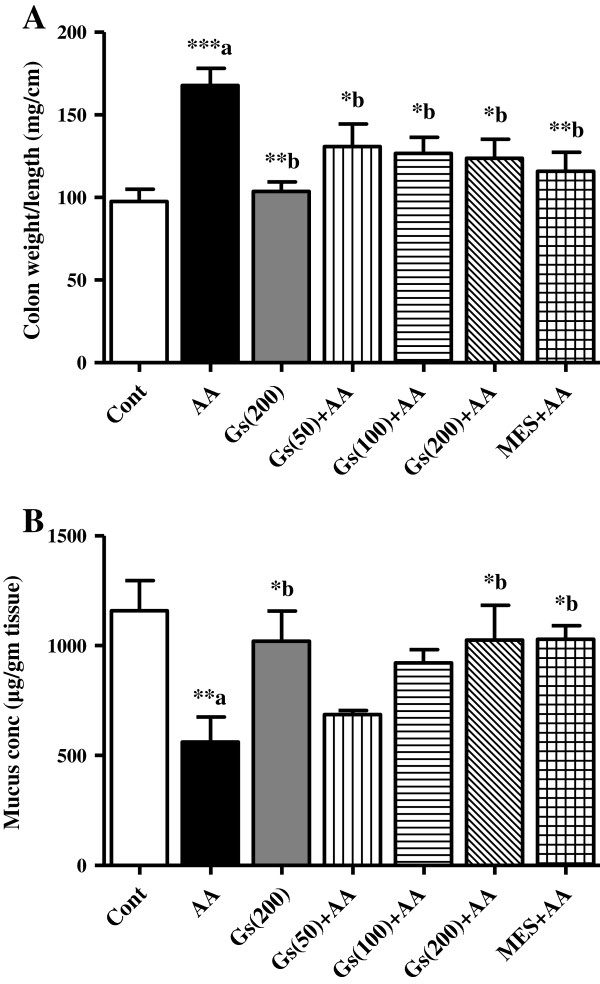
**Effect of *GS *(Gs) on [A] colon weight/length and [B] mucus content of rats in AA-induced UC.** Data are expressed as mean ± SEM (n = 6) and analyzed using one-way ANOVA followed by Student-Newman-Keuls multiple comparisons test. The statistical significance was considered as ^*^P < 0.05, ^**^P < 0.01 and ^***^P < 0.001 where ^‘a’^ compared with control and ^‘b’^ compared with AA.

In AA group, mean colonic weights were significantly (P < 0.001) increased compared to control animals. In seven days pretreatment with different doses of *GS* groups showed marked reduction in mean colon weights compared to AA group (Figure 1-A). Mucus content of rats' colon in AA group was significantly (P <0.01) reduced compared to controls. Only in higher dose of *GS* and MES groups, mucus content was significantly (P <0.05) increased when compared to AA group (Figure 1-B).

### Effect of *GS* on TBARS and sulfhydryl groups

**Figure 2:**
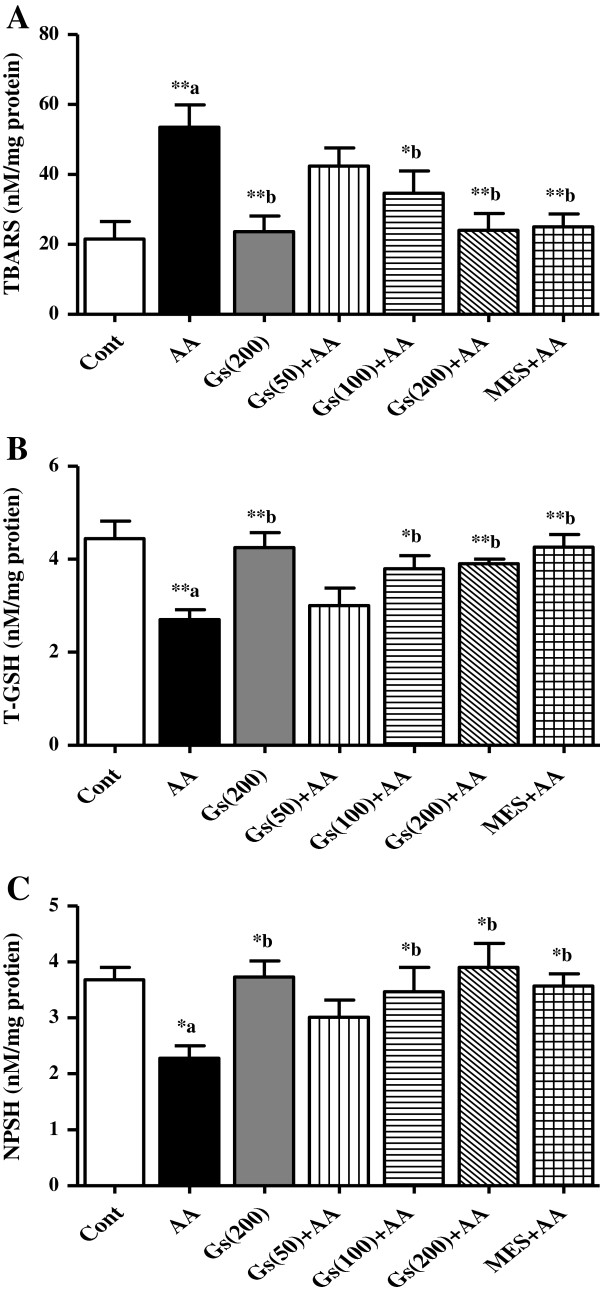
**Effect of *GS *(Gs) on colonic level of [A] TBARS, [B] T-GSH and [C] NPSH of rats in AA induced UC.** Data are expressed as mean ± SEM (n = 6) and analyzed using one-way ANOVA followed by Student-Newman-Keuls multiple comparisons test. The statistical significance was considered as ^*^P < 0.05, ^**^P < 0.01 and ^***^P < 0.001 where ^‘a’^ compared with control and ^‘b’^ compared with AA.

The TBARS levels were found significantly (P < 0.01) increased in AA administered group compared to control rats. Pretreated with higher two doses of and MES rats showed inhibition in TBARS levels compared to AA group (Figure 2-A). Sulfhydryl’s either in form of T-GSH or NP-SH significantly inhibited (P <0.01 and P <0.05, respectively) in colon tissues of AA group of animals. In pretreatments of rats with *GS* 100 mg/kg and 200 mg/kg ameliorated the reduced levels of T-GSH (P <0.05 and P <0.01, respectively) and NP-SH (P <0.05) compared AA group. Similarly, MES pretreatment also showed significant increase in T-GSH and NP-SH levels (P <0.001 and P <0.05, respectively) (Figure 2-B and 2-C).

### Effect of *GS* on SOD and CAT activities in colon tissues

**Figure 3:**
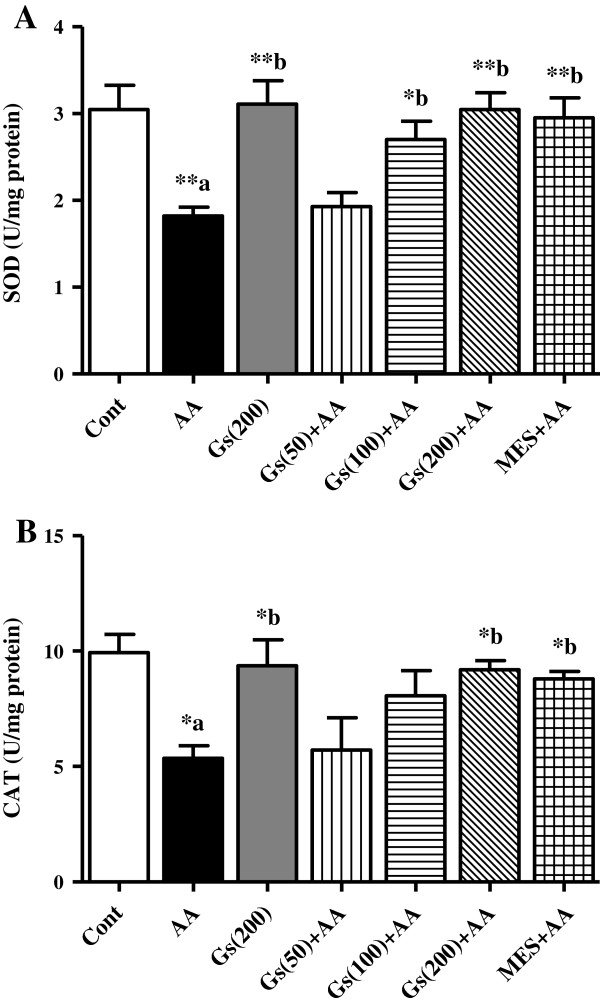
**Effect of *GS *(Gs) on colonic activities of [A] SOD and [B] CAT of rats in AA induced UC.** Data are expressed as mean ± SEM (n = 6) and analyzed using one-way ANOVA followed by Student-Newman-Keuls multiple comparisons test. The statistical significance was considered as ^*^P < 0.05, ^**^P < 0.01 and ^***^P < 0.001 where ^‘a’^ compared with control and ^‘b’^ compared with AA.

Activities of the antioxidant enzymes SOD and CAT were significantly (P <0.01 and P <0.05, respectively) inhibited in the colons of AA administered rats compared to control animals. Pretreatment with higher doses (100, 200 mg/kg) of *GS* showed marked increase in SOD activity (P <0.05 and P <0.01, respectively) compared to AA group (Figure 3-A). Although, only the higher dose (200 mg/kg) was able to enhance CAT activity (P <0.05) when compared to AA group (Figure 3-B). Pretreatment with MES produced marked increase in SOD and CAT activities (P <0.01 and P <0.05, respectively) compared to AA group (Figure 3-A and 3-B).

### Effect of *GS* on nucleic acids and TP levels in colon tissues

**Figure 4:**
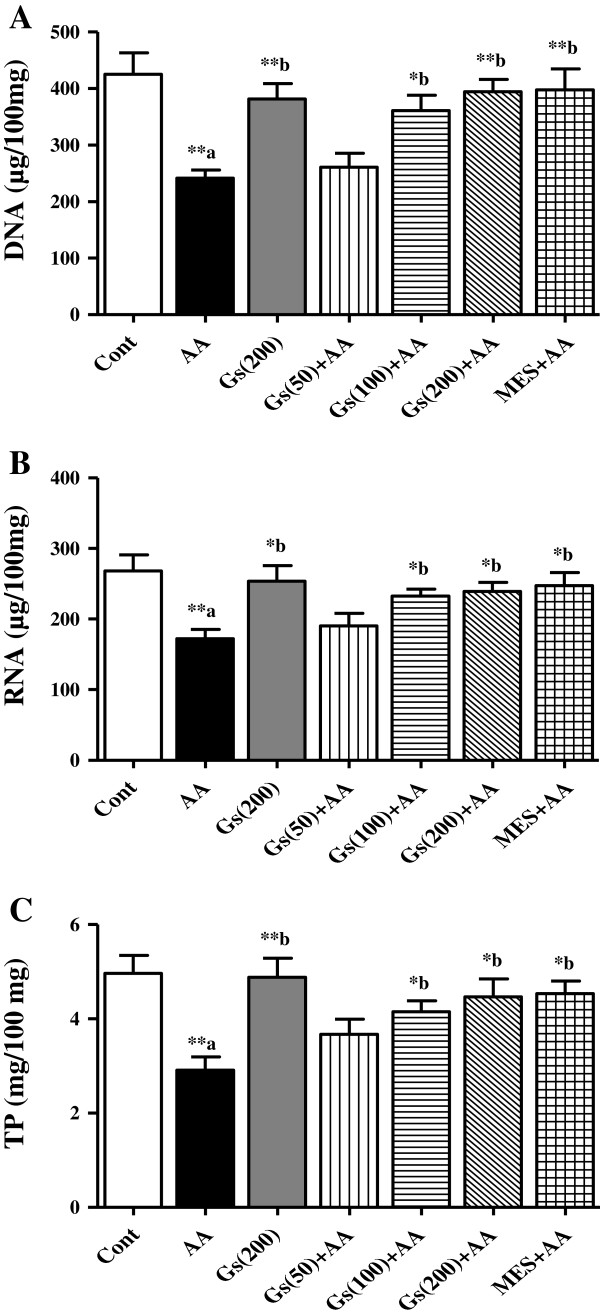
**Effect of *GS *(Gs) on colonic conc of [A] DNA, [B] RNA and [C] TP of rats in AA induced UC.** Data are expressed as mean ± SEM (n = 6) and analyzed using one-way ANOVA followed by Student-Newman-Keuls multiple comparisons test. The statistical significance was considered as ^*^P < 0.05, ^**^P < 0.01 and ^***^P < 0.001 where ^‘a’^ compared with control and ^‘b’^ compared with AA.

Nucleic acids (DNA and RNA) and TP levels in colon tissues were significantly (P <0.01) inhibited in AA administered group compared to control animals. In pretreated groups, with *GS* showed an increase in the DNA, RNA and TP levels when compared to AA group in a dose dependent manner and these levels were significantly increased in MES pretreated animals (Figures 4-A, 4-B and 4-C).

### Effect of *GS* on pro-inflammatory cytokines in colon tissues

**Figure 5:**
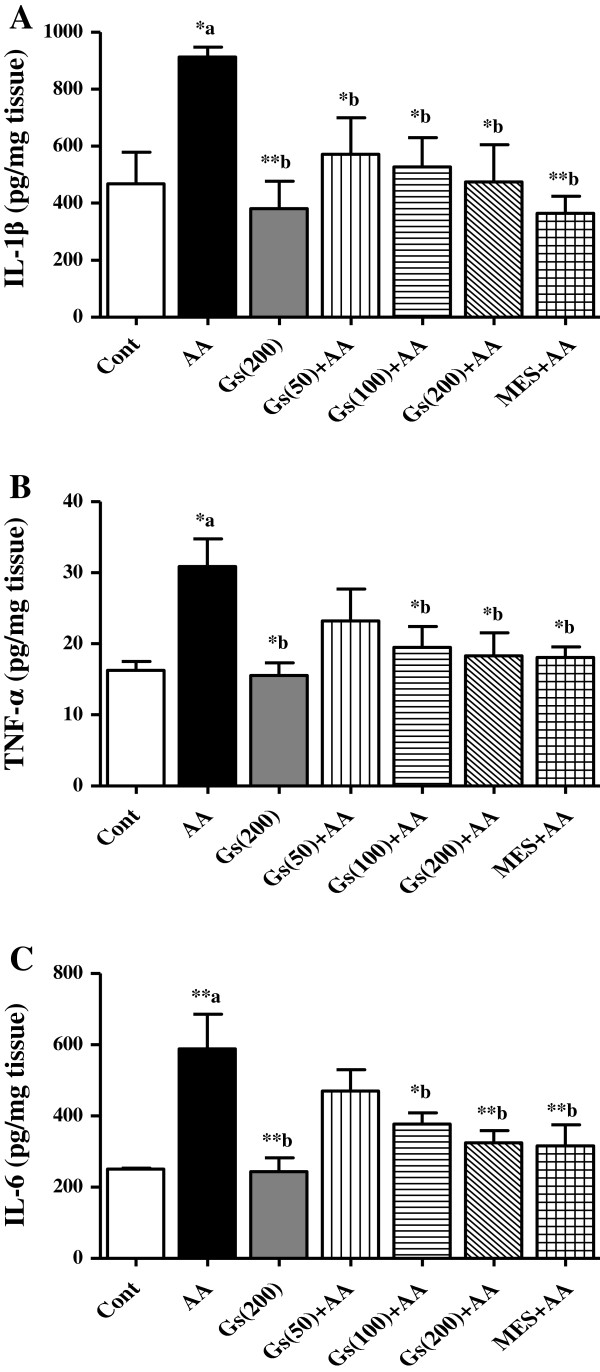
**Effect of *GS *(Gs) on colonic level of [A] IL-1β, [B] TNF-α and [C] IL-6 of rats in AA induced UC.** Data are expressed as mean ± SEM (n = 6) and analyzed using one-way ANOVA followed by Student-Newman-Keuls multiple comparisons test. The statistical significance was considered as ^*^P < 0.05, ^**^P < 0.01 and ^***^P < 0.001 where ^‘a’^ compared with control and ^‘b’^ compared with AA.

Colonic levels of pro-inflammatory cytokines IL-1β, IL-6 and TNF-α were significantly (P < 0.05, P < 0.05 and P < 0.01 respectively) increased in AA administered group compared to control rats. In all *GS* pretreated groups a significant (P <0.05) lower levels of IL-1β was found (Figure 5A). While 100 mg/kg and 200 mg/kg *GS* doses groups had only significant reduction in TNF-α (P <0.05) and IL-6 (P <0.05 and P <0.01, respectively) when compared to the animals in AA group. However, MES pretreatment showed significant decrease in all these cytokines levels when compared to AA group (Figure 5-A, 5-B and 5-C).

### Effect of *GS* on PGE_2_ and NO levels in colon tissues

**Figure 6:**
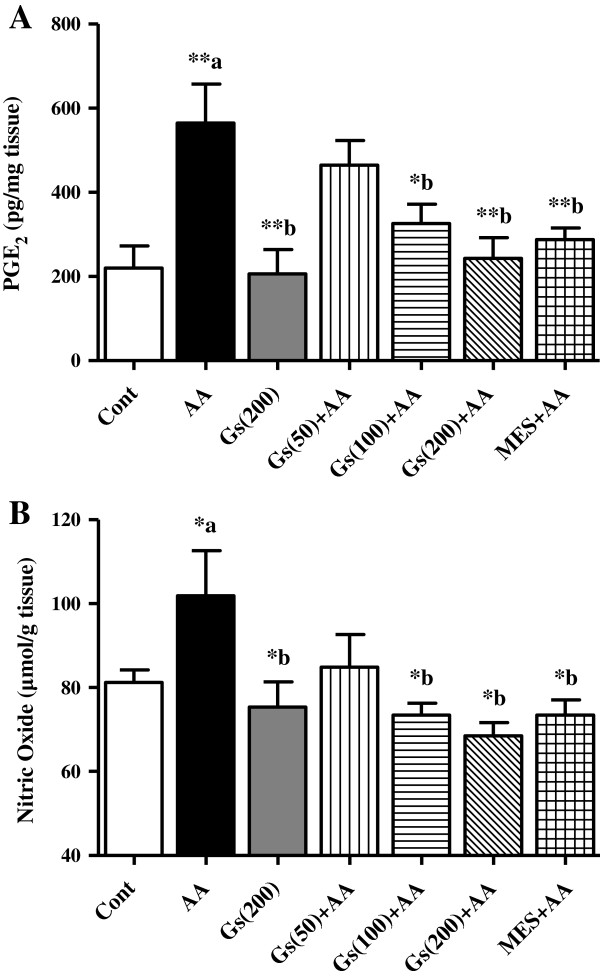
**Effect of *GS *(Gs) on colonic level of [A] PGE**_**2 **_**and [B] NO of rats in AA induced UC.** Data are expressed as mean ± SEM (n = 6) and analyzed using one-way ANOVA followed by Student-Newman-Keuls multiple comparisons test. The statistical significance was considered as ^*^P < 0.05, ^**^P < 0.01 and ^***^P < 0.001 where ^‘a’^ compared with control and ^‘b’^ compared with AA.

In addition there was a marked elevation in PGE_2_ and NO levels in colon tissues of AA administered group when compared to control (P <0.01 and P <0.05, respectively). *GS* at (100 and 200 mg/kg) the elevated PGE_2_ levels were significantly inhibited (P <0.05, P <0.01 respectively). The level of PGE_2_ was also inhibited in MES compared to AA groups (Figure 6-A). Similarly, NO levels in colon tissues were reduced significantly (P <0.05) in *GS* pretreated rats (100 and 200 mg/kg) to AA alone. MES pretreated groups also showed significant reduction in NO levels compared to AA administered animals (Figure 6-B).

### Effect of *GS* on histopathological changes in colon tissues

**Figure 7:**
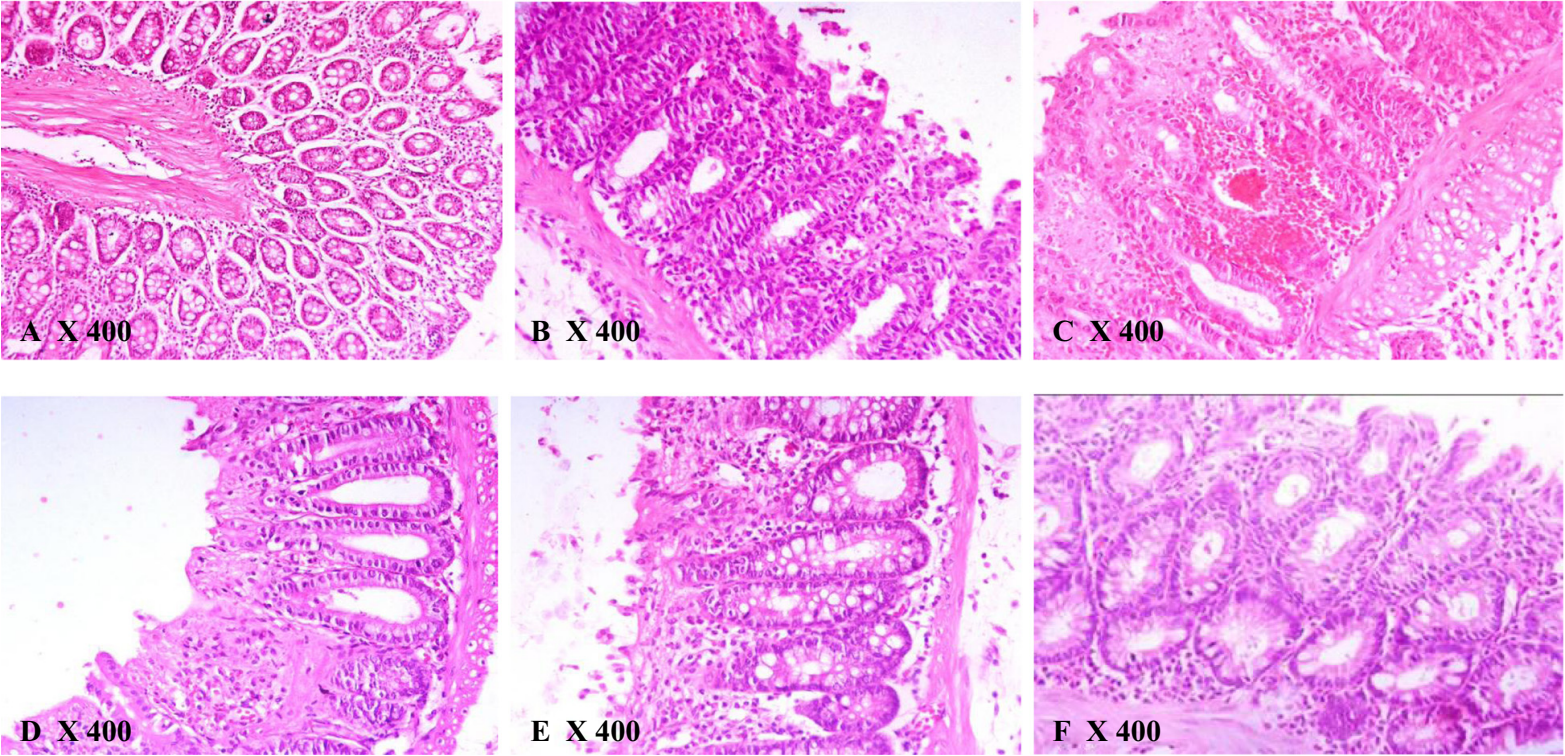
**Histopathological sections of colons from rats stained with H&E (400X).** Colonic microscopic image of **[A]** Normal rat colon from Cont group with intact mucosal layer and epithelial; **[B]** AA treated rat colon with diffused active colitis, extensive damage including edema in submucosa and chronic inflammatory cells infiltrate with widely ulcerating mucosa, and hemorrhages; **[C, D & E]** dose dependent reparative epithelial changes and ulcer healing with lymphoid follicle in colon of *GS* treated rats (50, 100 and 200 mg/kg , respectively); **[F]** attenuated cell damage with complete ulcer healing in MES treated group.

As shown in Figure 7, histopathological screening of rat’s colon cross sections revealed normal looking mucosal epithelium with no necrosis or inflammation in the control group (Table 2 and Figure 7-A). In AA group (Figure 7-B), a diffused active UC was seen along with severe necrosis and inflammation associated with edema, goblet cell hyperplasia (Table 2 and Figure 2-B). Pretreatment with *GS* (50 mg/kg) resulted in a slight healing of epithelial cells ulceration with moderate degree of necrosis and inflammation and less goblet cells (Table 2 and Figure 7-C). However histopathological assessment of colon in *GS* (100) + AA group revealed more healing of the mucosal epithelium with less eroded surface surrounded by fewer inflammatory edema and less necrosis (Table 2 and Figure 7-D). Pretreatment with the higher dose of *GS* (200 mg/kg) resulted in a total healing of the superficial eroded mucosa with little hemorrhage, edema and necrosis and fewer inflammatory cells infiltrate and goblet cells (Table 2 and Figure 7-E). Colon tissues of animals pretreated with MES (300 mg/kg) as a standard drug showed markedly healed and improved intestinal mucosa that can be compared to colon section from control animals (Table 2 and Figure 7-F).

**Table 2:** **Effect of *GS *** **on histopathological changes of colonic tissues of rats with AA-induced UC**

	**Ulceration**	**Hyperemia**	**Necrosis**	**Edema**	**Cellular Infiltrate**	**Goblet Cell Hyperplasia**
**Cont**	0	0	0	0	0	0
**AA**	3	3	4	3	4	2
**GS(50) + AA**	2	2	2	2	2	1
**GS(100) + AA**	1	1	1	1	2	1
**GS(200) + AA**	0	1	0	1	1	0
**MES + AA**	0	1	0	1	1	0

0: No abnormality detected;

1: Damage or active changes < 25%;

2: Damage or active changes < 50%;

3: Damage or active changes < 75%;

4: Damage or active changes > 75%.

## Discussion

In the present study the preventative properties of *GS* leaves extract against experimentally induced model of IBD in Wistar rats were investigated. The histopathological assessment revealed that pretreatment with preserved the functional cytoarchitecture of the entire colonic mucosa, congestion, ulceration, erosions, necrosis and inflammation caused by AA in a dose-dependent manner. Moreover, *GS* leaves extract markedly protected the colonic mucosal content and prevented oxidative and inflammatory response in the colon of AA induced rats.

Experimentally induced UC by 4% AA is a well-recognized model for IBD. The colonic changes following rectal application of AA to rodents are characterized by mucosal ulceration, hemorrhage and inflammation, which are similar to IBD in human [30]. It also causes infiltration of leukocytes to the damaged area and rupture of colonic barrier, along with an inflammatory mediator’s release, including cytokines and arachidonic acid metabolites as well as release of ROS, leading to oxidative damage [8,9]. In the current investigation, rectal application of AA significantly increased animals colon weights, which was associated with severe tissue ulceration, necrosis, goblet cell hyperplasia and inflammatory infiltrate as demonstrated in the histopathological screening, which are in accordance with earlier reports using the same animal model [21,31]. Defects in the colonic mucosal barrier functions are among the etiological factors that characterize IBD [32]. In the current study, the protective colonic mucus content was markedly altered by AA, which is in agreement with the study by Popov et al. 2006 [24]. The mucus layer is well known to enhance the repair of the chemically damaged epithelium [33].

Several therapies have been used in the management of IBD. However, their adverse effects and toxicity represent major clinical problem [34]. Therefore, naturally occurring alternative options has been suggested along with the conventional therapies [35]. Our previous work demonstrated that *GS* leaves extract effectively protected against chemically induced gastric ulcers [33]. Results of the present study showed that the increased colon weight after AA administration was significantly reduced by the pretreatment of the animals with *GS*, indicating a decreased colon inflammation which was demonstrated by histopathological assessments. Pretreatments with *GS* inhibited colonic wall mucus depletion in the UC rat model, which could be attributed to its anti-inflammatory property, similar to our previous studies showing attenuation of the gastric mucosal damage-induced by absolute ethanol [33].

Both the reported forms of IBD are multi-factorial intestinal inflammatory disease, however, pro-inflammatory mediators is considered to play a crucial role in the pathogenesis of IBD [36]. They can modulate mucosal immune system, by the alteration of epithelial integrity and colon injury by infiltration of the neutrophils and macrophages [37]. Migration of granulocytes and other leukocytes to the inflamed mucosa and superficial ulcers results in overproduction of pro-inflammatory cytokines [8,9]. In both IBD forms, levels of pro-inflammatory cytokines, such as IL-1β, TNF-α, and IL-6, were found to be increased [38-40], which suggest that these inflammatory mediators are engaged in determining the severity of the disease. In the present study, pro-inflammatory cytokines including IL-1β, TNF-α, and IL-6 were significantly elevated in colon tissues in AA administered group, suggesting a role of inflammation in the pathogenesis of the disease which is supported by the histopathological results showing epithelial cell necrosis, edema, and neutrophil infiltration in the tissue. Our findings are in agreement with earlier experimental and clinical data reported by others in a number of studies [24,31,41,42]. Next, we found increased colonic levels of PGE_2_ and NO in AA group of animals, which is in accordance with other investigations [43,44]. This increase in the levels of inflammatory molecules may be mediated through pro inflammatory cytokines. The anti-inflammatory properties of *GS* leaves extract were reported previously using various inflammatory animals models [45,46]. *GS* leaves extract was found to reduce the level of pro-inflammatory cytokines (IL-1β, IL-6 and TNF-α) in AA colon tissue. The level of prostaglandins and NO is regulated by the cellular enzymes COX-2 and iNOS, respectively. These enzymes are known to be enhanced by inflammatory mediators during the burden of UC [47,48]. Therefore, we suggest the anti-inflammatory effect of *GS* might be mediated through inhibiting the level PGE_2_ and NO in AA model of UC.

Oxidative stress is well known to play the major role in the pathophysiology of IBD [49,50]. Induction of UC in experimental animals causes oxidative injury due to imbalance between the levels of pro-oxidant and antioxidant systems [51]. UC is characterized by overproduction of reactive oxygen and nitrogen species leading to significant cellular adverse effects such as LPO and damage to tissue proteins and nucleic acids [10,52]. Furthermore, increased levels of free radicals were found in colonic tissue specimens of patients with UC [53,54]. Antioxidant enzymes such as SOD and CAT, and the non-enzymatic sulfhydryl groups play the major role in the organism defense against excess free radicals generated under disease conditions [55]. In the present investigation, concentrations of protein and non-protein sulfhydryl groups as well as activities of the antioxidant enzymes such as SOD and CAT were severely reduced in the colon following AA administration, which clearly indicates increased level of oxidative stress which may damage cells by lipid peroxidation of membranes and oxidation of cellular proteins. Indeed, increased levels of TBARS and free radicals found in the study may damage cells as observed by histopathological investigations.

Another damaging effect of oxidative stress have been noted in the present study, is in the alteration in the levels of nucleic acids and proteins in the colon of AA treated animals. These observations were also previously reported by others, which confirm the oxidative damages to cellular macromolecules thereby may weaken epithelial cellular integrity and delay colonic mucosal healing [5,31]. Thus, it is suggested that those substances that prevent free radicals production or potentiate the endogenous enzymatic or non-enzymatic antioxidant system can have beneficial effects in ulcerative colitis. In agreement with previous studies [56,57], pretreatment with *GS* increased antioxidant status and lowered LPO in AA model of UC which suggests its colonic protective effect by enhancing of nucleic acids and proteins levels. It is well known that *GS* has anti-diabetic properties with antioxidant activity [58]. Previously antioxidant and anti-LPO effects of *GS* were reported in several animal models [59,60] and also in an *in vitro* study by Rachh and colleagues, 2009 [61]. Several pharmacological studies have demonstrated that the antioxidant properties of *GS* are mainly through the major bioactive constituents in its leaves, which are a group of oleanane type triterpenoid saponins known as gymnemic acids [12,16], alkaloids, acidic glycosides and anthroquinones and their derivatives [17]. These constituents were also found in our preliminary phytochemical analysis from the *GS* leaves extract.

## Conclusion

Findings of the current investigation represent a clear evidence of the preventative ability of *GS* leaves extract against the damage in the experimentally induced UC by AA in Wistar rats. These protective effects could be attributed to the powerful anti-inflammatory and antioxidant properties present in the bioactive constituents of *GS*. The outcomes of present study may implicate in future clinical trials of the *GS* leaves extract or its bioactive constituents as natural, safe and effective treatments for patients with IBD.

## Competing interests

The authors declare that they have no competing interests.

## Authors’ contributions

AMA, HMA and MYP have performed experimental designed, induction of colitis and animal treatment. HMA, MMA, MYP and SSA have carried out biochemical and statistical analysis as well as interpretation of the data. AMA participated with MSO in histopathological investigation and writing of the manuscript. SSA and MMA has revised and submitted the final manuscript. All authors read and approved the final manuscript.

## Pre-publication history

The pre-publication history for this paper can be accessed here:

http://www.biomedcentral.com/1472-6882/14/49/prepub
